# CRLF1 and CLCF1 in Development, Health and Disease

**DOI:** 10.3390/ijms23020992

**Published:** 2022-01-17

**Authors:** Laura Crisponi, Insa Buers, Frank Rutsch

**Affiliations:** 1 Institute of Genetic and Biomedical Research (IRGB), National Research Council, 09042 Monserrato, CA, Italy; 2 Department of General Pediatrics, Muenster University Children’s Hospital, Albert-Schweitzer-Campus 1, Gbde. A1, D-48149 Münster, Germany; insa.buers@ukmuenster.de; 3 Center for Rare Diseases, Muenster University Hospital, D-48149 Münster, Germany

**Keywords:** CRLF1, CLCF1, type I cytokine receptor, development, Crisponi/cold-induced sweating syndrome

## Abstract

Cytokines and their receptors have a vital function in regulating various processes such as immune function, inflammation, haematopoiesis, cell growth and differentiation. The interaction between a cytokine and its specific receptor triggers intracellular signalling cascades that lead to altered gene expression in the target cell and consequent changes in its proliferation, differentiation, or activation. In this review, we highlight the role of the soluble type I cytokine receptor CRLF1 (cytokine receptor-like factor-1) and the Interleukin (IL)-6 cytokine CLCF1 (cardiotrophin-like cytokine factor 1) during development in physiological and pathological conditions with particular emphasis on Crisponi/cold-induced sweating syndrome (CS/CISS) and discuss new insights, challenges and possibilities arising from recent studies.

## 1. Introduction

### 1.1. Cytokines and Specific Receptors

Cytokines are a broad category of small secretory proteins (<40 kDa) produced by a wide range of cells in response to different stimuli and released for cell signalling. They play vital regulatory roles in diverse processes such as immune function, inflammation, haematopoiesis, cell growth and differentiation. Each cytokine has a matching cell-surface receptor. Their interaction in the extracellular environment elicits intracellular signalling cascades leading to altered gene expression in the target cell and consequent biological effects, such as differentiation, proliferation, and activation of the target cell. Their action may occur in an autocrine (acting on the same cell), paracrine (acting on nearby cell), or endocrine (acting on distant cell) manner. Cytokines show different properties such as pleiotropy (different effects on different types of target cells), redundancy (multiple cytokines have the same effect), or cascade action (one cytokine stimulates its target cells to make additional cytokines). Furthermore, they can act synergistically (combined effect of two cytokines on cellular activity is more potent than the effect of individual cytokine) or antagonistically (inhibition of one cytokine’s effect by another). A significant challenge when discussing cytokines is their complex classification. Classification based on their function, cell of secretion, or target of action is unsuitable considering their redundancy and pleiotropy. Instead, the current tendency is to classify cytokines according to the type of receptor they bind to. The assignment between cytokines and receptors is based on their structural domains. Based on sequence homologies and structural features, cytokine receptors are classified into five prominent families: type I cytokine receptors, type II cytokine receptors, immunoglobulin superfamily receptors, tumour necrosis factor (TNF)-like receptors, and chemokine receptors [1].

### 1.2. Type I Cytokine Receptors

The type I cytokine receptor family is also known as the hematopoietin receptor family since most of the cytokine receptors effective in the hematopoietic system belong to this family. It is the largest family with more than 50 members, including receptors for erythropoietin (EPO), prolactin (PRL), growth hormone (GH), thrombopoietin (TPO), leptin (LEP), granulocyte-macrophage colony-stimulating factor (GM-CSF), leukaemia inhibitory factor (LIF), interleukin-3 (IL-3), IL-5, IL-7, and IL-6 [2,3].

**Figure 1:**
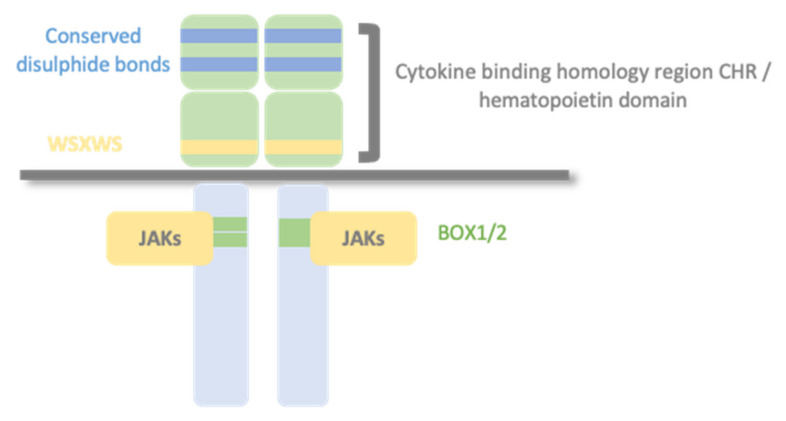
Type I cytokine receptors. Type I cytokine receptors consist of multiple (usually two) transmembrane protein chains modules, similar in their basic structure. Each chain possesses an extracellular cytokine binding homology region (CHR) also called the hemopoietin domain, involved in cytokine interaction and a cytoplasmic domain involved in signal transduction. Structurally, the hematopoietin domain comprises fibronectin type III repeats with two pairs of disulfide-linked cysteines and a highly conserved WSXWS motif (X indicates any amino acid). The cytoplasmic domain contains proline-rich Box1/Box2 motifs, which mediate interaction and activation of Janus tyrosine kinases (JAKs).

Type I cytokine receptors recognize and respond to type I cytokines and consist of multiple (usually two) transmembrane protein chain modules, similar in their basic structure. Each chain possesses an extracellular domain, also called the hemopoietin domain, involved in ligand/cytokine interaction and a cytoplasmic domain involved in signal transduction. Structurally, the hematopoietin domain consists of two fibronectin type III repeats domains at approximately a 90° angle, forming the cytokine binding homology region (CHR), with two pairs of disulfide-linked cysteines and a highly conserved WSXWS motif (where X indicates any amino acid; [3,4]). The cytoplasmic domain contains proline-rich Box1/Box2 motifs [5]. Cytokine receptors can have additional extracellular domains besides CHR, exerting different roles: i.e., correctly orienting the receptor to activate intracellular kinases, facilitating cytokine binding, or modulating intracellular trafficking to the membrane [6] (Figure 1). The primary mechanism for receptor activation is that the binding of the cytokine to the CHR domains leads to dimerization of the receptor ectodomains [7]. Some type I cytokine receptor family members are homodimers when bound to their activating ligand. In contrast, other receptor members are heterodimers, with two or three protein chains, one of them shared by different cytokine receptor complexes.

There are three typical class I cytokine receptor protein chains: Glycoprotein 130 (GP130), common beta chain (βc), and common gamma chain (γc) [1]. The sharing of cytokine receptor chains and signalling pathways provides a mechanism for functional redundancy. The existence of more than one receptor for that cytokine can explain the pleiotropic effects of a single cytokine [8], while the expression and distribution of cytokine receptors among the different cell types determine the specificity of cytokine activity [9]. The combinatorial array of cell type-specific and context-specific expression of cytokines and receptor complexes is responsible for various host protective immunity, organ development and metabolism, haematopoiesis, tumorigenesis, development, and inflammation [10].

Aside from membrane-bound cytokine receptors, soluble variants of several cytokine receptors are generated by different molecular mechanisms. A membrane-anchored cytokine receptor can be cleaved by a protease, resulting in a soluble receptor; a differential mRNA splicing can lead to a transcript encoding for a soluble form of the cytokine receptor; a soluble cytokine receptor can be released from cells via exosomes. These soluble receptors modulate the function of cytokines in several ways. They can act as agonistic and antagonistic decoy receptors, forming active receptor/cytokine complexes that can activate cells or compete with their membrane-bound counterparts for the ligand [9].

### 1.3. Glycoprotein 130 Cytokine Receptor Family and IL-6 Cytokines

Glycoprotein 130 (GP130) is one of the shared type I cytokine receptor chains and the standard signal-transducing molecule for almost all cytokines of the IL-6 family. The IL-6 family of cytokines comprises 12 members, namely IL-6, IL-11, IL-27, Oncostatin M (OSM), leukaemia inhibitory factor (LIF), ciliary neurotrophic factor (CNTF), cardiotrophin-1 (CT-1), cardiotrophin-like cytokine factor 1 (CLCF1), neuropoietin, IL-31, IL-35 and IL-39 [11]. All IL-6 family cytokines, apart from IL-31, use at least one GP130 β-receptor subunit (GP130) as part of the signal-transducing complex [11]. IL-6 family cytokines transduce their signals through GP130 homodimers or GP130-containing heterodimers. No natural cytokine can activate GP130 in the absence of other receptor chains. Non-signalling α-receptors (IL-6Rα, IL-11Rα, and CNTFRα), and signal-transducing receptors (LIFRβ and OSMR) are other receptor chains involved in recognition of the IL-6 family cytokine. Glycoprotein 130 associates with Janus tyrosine kinases (JAK1, JAK2), and tyrosine kinase 2 (TYK2), activating signal transducer and activator of transcription STAT1 and STAT3 [12]. A specific receptor combination profile characterizes each IL-6-type cytokine that, in all cases, involves at least one molecule of GP130. IL-6, IL-11 and CNTF first bind specifically to their respective α-receptor subunits. Only this complex then efficiently recruits the signalling receptor chains. IL-6 and IL-11 are the only IL-6-type cytokines that signal via GP130 homodimers. The remaining IL-6 type cytokines signal via heterodimers of either GP130 and the LIFRβ (LIF, CNTF, CT-1 and CLCF1) or GP130 and the OSMR (OSM). OSM can recruit two different receptor complexes, forming the heterodimers LIFRβ–GP130 and OSMR–GP130. LIF and OSM directly engage their signalling receptor chains without requiring additional α-receptor subunits [13]. GP130 is ubiquitously expressed [14], whereas all other IL-6 cytokines receptor chains show a more restricted expression pattern. Therefore, the expression of these second receptor chains determines the specific response of a cell to a given cytokine [15,16].

### 1.4. The JAK/STAT Pathway

**Figure 2:**
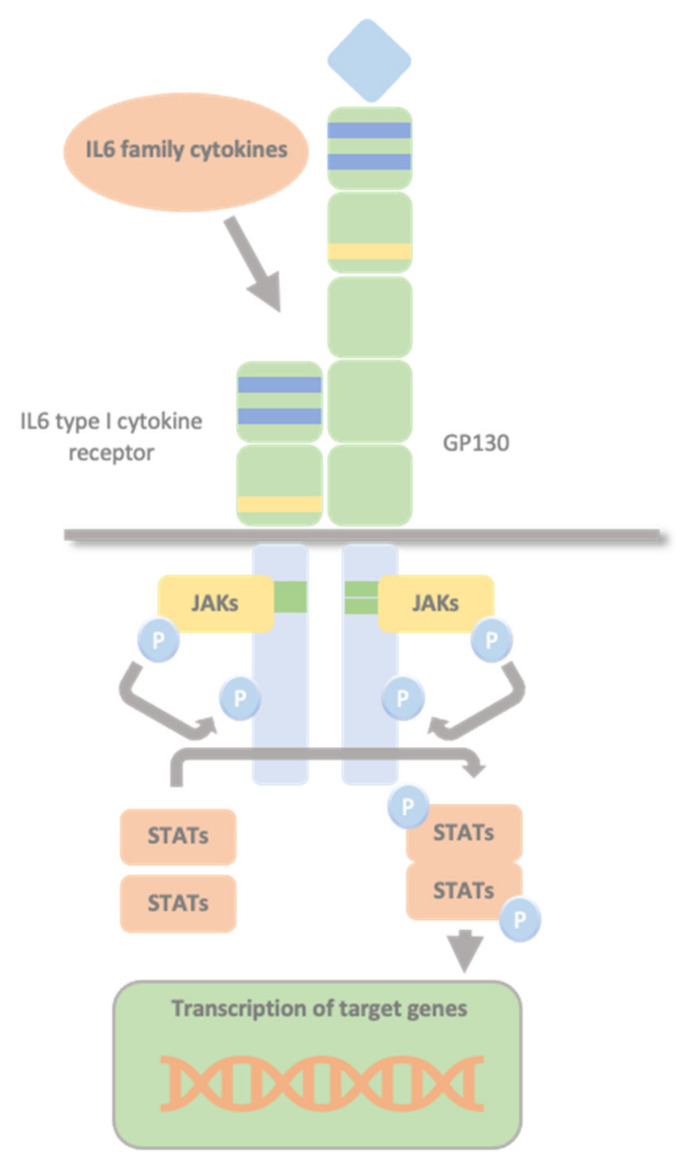
Glycoprotein 130 Cytokine Receptor Family and IL-6 Cytokines. IL-6 family cytokines use GP130 to transduce their signals through GP130 homodimers or GP130-containing heterodimers with the type I cytokine non-signalling α-receptors (IL-6Rα, IL-11Rα, and CNTFRα), and the signal-transducing receptors (LIFRβ and OSMR). The intrinsic Box1/2 motifs mediate the interaction and activation of JAK kinase. The activation consists of JAK auto- or transphosphorylation, that subsequently phosphorylates specific tyrosine residues in the intracellular domains of the receptor and subsequent phosphorylation of the receptor by JAKs. This creates docking sites for STAT molecules which, once bound to the receptor are also phosphorylated by JAKs. Phosphorylated STATs dissociate from the receptor, dimerize, and translocate to the nucleus, where they control transcription by directly binding to the DNA to mediate transcriptional program determining fundamental phenotypic changes in the cell.

Type I cytokine receptors lack intracellular kinase activity and rely on associated JAKs to transmit intracellular signalling. The binding of the cytokine to its cytokine receptor drives receptor dimerization, oligomerization, and conformational changes that facilitate the activation of the JAKs, constitutively bound to these receptors’ intracellular domains. The intrinsic Box1/2 motifs mediate the interaction and activation of JAK kinase. The activation consists of JAK auto- or transphosphorylation, which subsequently phosphorylate specific tyrosine residues in the intracellular domains of the receptor and subsequent phosphorylation of the receptor by JAKs. This modification creates docking sites for STAT molecules which, once bound to the receptor, are also phosphorylated by JAKs [1,17,18]. Phosphorylated STATs dissociate from the receptor, dimerize, and translocate to the nucleus. They control transcription by directly binding to the DNA to mediate transcriptional programs determining fundamental phenotypic changes in the cell (Figure 2).

The JAK/STAT pathway is tightly controlled through the induction of suppressor of cytokine signalling (SOCS) proteins, which subsequently inhibit STAT-mediated signal transduction. SOCS1 and SOCS3 are the most widely characterized members. They comprise an N-terminal domain, including a kinase inhibitory region (KIR), a central SH2 domain and a C-terminal SOCS box domain. They act as classical feedback inhibitors by three distinct mechanisms: kinase inhibition (of JAK), binding-site competition (of STATs) and proteasomal degradation of receptor complexes [19].

In addition, type I receptors activate signalling pathways and transcription factors other than the JAK/STATs, MAPK, and PI3K pathways. These pathways connect activation of the receptor complexes directly to the transcription of genes to control different processes, such as control of cell growth, differentiation, maturation, and apoptosis. Dysregulation of cytokine signalling results in human diseases and pathology. Knowledge of the mechanisms regulating cytokine receptor signalling is essential for understanding both potential pathological consequences and the development of potential therapeutic targets [19,20].

This review summarizes the current knowledge on the soluble type I cytokine receptor CRLF1 (cytokine receptor-like factor-1) and the IL-6 cytokine CLCF1 (cardiotrophin-like cytokine factor 1), focusing on their role in various processes. We describe their effects in development, in physiological and pathological states, focusing on Crisponi/cold-induced sweating syndrome (CS/CISS) and discuss new insights, challenges, and opportunities resulting from recent studies on these cytokines.

## 2. Overview of Cardiotrophin-like Cytokine Factor 1/Cytokine Receptor-like Factor-1

### 2.1. CLCF1 Key Concepts

Cardiotrophin-like cytokine factor 1 (CLCF1) is a member of the IL-6 family and was firstly identified in 1999 [21,22]. *CLCF1* consists of three exons and maps to 11q13.2. Its cDNA (RefSeq NM_013246) encodes for a 225 aa protein (RefSeq NP_037378) with a conventional signal peptide spanning from amino acid 1 to 27. The mature form consists of a 198 aa peptide of 22 kDa. Human and murine CLCF1 share a 96% amino acid identity [21]. In addition to features typical of IL-6 family cytokines, including neurotropic effects, it shows B cell-stimulating capability. Therefore, it was named novel neurotrophin-1yB cell-stimulating factor-3 [21] or cardiotrophin-like cytokine (CLC) [22], according to its high similarity with cardiotrophin-1 (CT-1), another member of the IL-6 family. It is expressed mainly in lymphoid tissue, such as lymph nodes and spleen, in humans and mice, indicating relevance to the immune system. However, it is also present in various organs, like most IL-6 family members. This finding may indicate a functional pleiotropy, a characteristic of cytokines in general.

### 2.2. CRLF1 Key Concepts

The human *CRLF1* gene consists of nine exons and maps to 19p13.11. Its cDNA (RefSeq NM_004750) encodes a 422 aa precursor protein of 46 kDa (RefSeq NP_004741) with a 37 amino acid putative signal peptide. Human and murine CRLF1 share a 96% amino acid identity [23]. It belongs to the cytokine type I receptor family, containing a hematopoietin domain with a pair of fibronectins (FBN) type III modules and a highly conserved WSXWS motif. This extremely high level of conservation among vertebrates during recent evolution, as also present in CLCF1, indicates an important functional role for both. The expression pattern of human and mouse *CRLF1* suggests a role for the protein in the immune system and foetal development. Human *CRLF1* mRNA is predominantly expressed in the adult spleen, thymus, lymph node, appendix, bone marrow, stomach, placenta, heart, thyroid, ovary, fibroblasts, and foetal lung. It is up-regulated by pro-inflammatory cytokines such as TNF-a, IL-6, and IFN-g, suggesting that hCRLF1 may be involved in regulating the immune system during an inflammatory response [23]. Whole-mount in situ hybridization in mice embryos at different time points in the developing embryo revealed *Crlf1* expression at multiple sites starting from E9.5 [24].

### 2.3. CLCF1/sCNTFRα Complex and CRLF1/CLCF1/CNTFRα Complex

Although CLCF1 contains a signal peptide, its secretion is inefficient unless co-expressed with its chaperones, both members of the type 1 cytokine receptor family, CRLF1 (cytokine receptor-like factor 1), a soluble cytokine receptor, or sCNTFRα (soluble ciliary neurotrophic factor receptor α) [25,26,27]. Studies revealed that CLCF1 and sCNTFRα should be complex, enough to be active, within an intracellular environment or on the cell surface [26].

**Figure 3:**
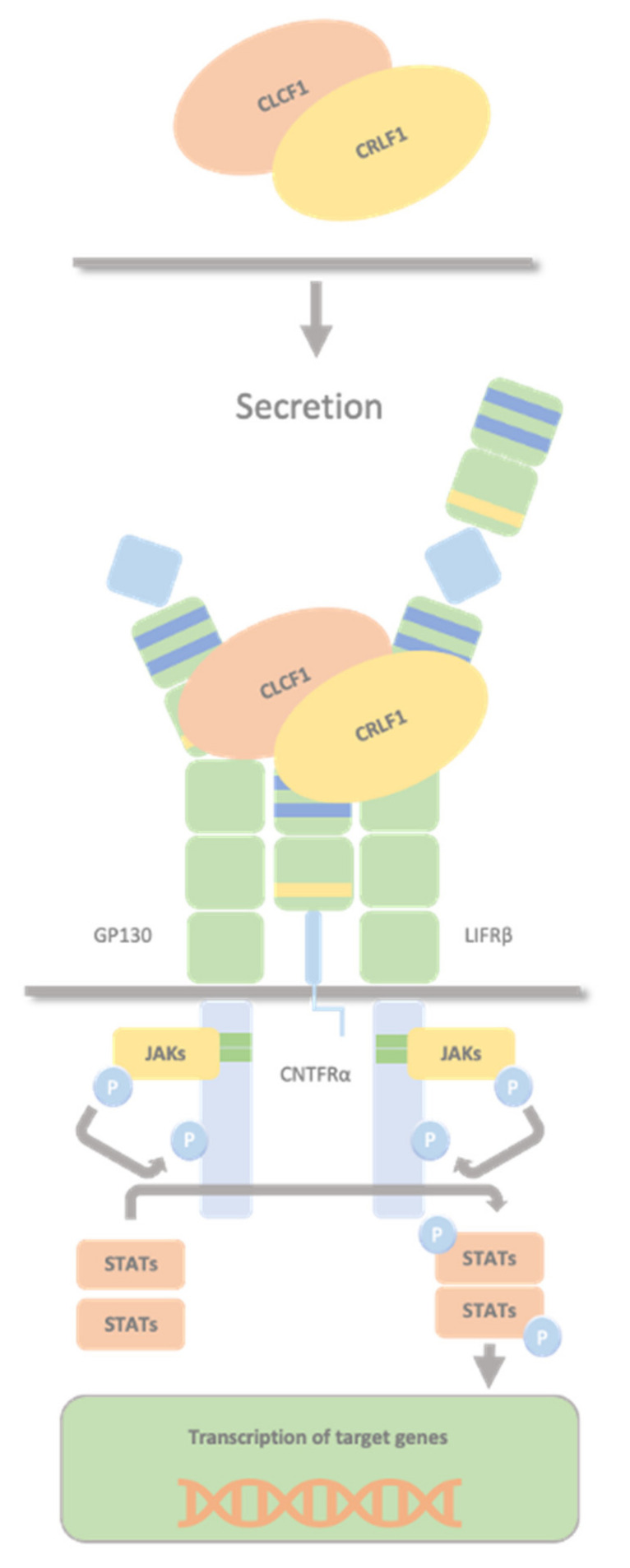
CRLF1/CLCF1/CNTFRα complex. The secreted heterodimer CRLF1/CLCF1 activates the CNTF receptor (a tripartite receptor complex formed by GP130, CNTFRα, and LIFRβ), with the induction of downstream signalling events, including activation of the JAK1/STAT3 pathway. Both CLCF1 and CRLF1 have a binding site for CNTFRα and interact with each other.

CLCF1 interact by two different sites with CRLF1 and CNTFRα (both the soluble and the membrane-bound subunit); site 1 for sCNTFRα [26] and site 3 for CRFL1 [28]. The most crucial residues in site 1 involved in the interaction with sCNTFRα are Trp67, Arg170, and Asp174. The residues in site 3, which are essential for the interaction with CRLF1, are Phe151 and Lys154, and the same site is necessary for the interaction with LIFRβ [28]. CLCF1 site 2 is essential for GP130 binding [28]. The secreted heterodimer CRLF1/CLCF1 activates the CNTF receptor (a tripartite receptor complex formed by CNTFRα, GP130 and LIFRβ), with the induction of downstream signalling events, including activation of the JAK1/STAT3 pathway (Figure 3). Although CRLF1 is not needed to activate the CNTF receptor, early secretion of the whole CRLF1 protein seems necessary for adequate CLCF1 secretion kinetics and proper activation of the CNTF receptor pathway, implying that CRLF1 is more than just a facilitator of CLCF1 secretion [29]. As CLCF1, CRLF1 also contains a site for CNTFRα binding and both bind to CNTFRα with moderate affinity. The CRLF1/CLCF1 complex, including two binding sites (one in each subunit), generates a higher affinity binding for CNTFR [30]. It is a more potent inducer of STAT3 phosphorylation than the individual CLCF1 subunit. It could be that the binding of CRLF1 with CLCF1 creates a more stable and optimal conformation for CLCF1, increasing the affinity for CNTFR [30].

### 2.4. CRLF1/CLCF1/CNTFRα/SorLA Complex

**Figure 4:**
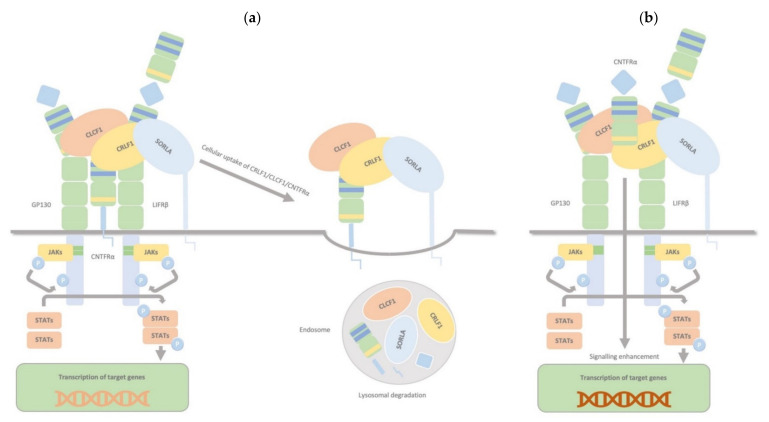
CRLF1/CLCF1/CNTFRα/SorLA complex. CRLF1 has at least three independent binding sites: one for CLCF1, one for CNTFRα, and one for SorLA, and it can bind all its targets simultaneously. The complexes CRLF1/CLCF1 and CRLF1/CLCF1/sCNTFRα bind to SorLA via the CRLF1 subunit, and this binding, according to its partners, can either (**a**) (membrane-bound subunit CNTFRα) modulate the cellular response to CRLF1/CLCF1 by mediating CRLF1-dependent endocytosis and subsequent lysosomal degradation or (**b**) (soluble form of CNTFRα) enhance signalling, notably in CNTFR-deficient cells, by binding and concentrating complexes of CRLF1/CLCF1/ sCNTFRα on the cell membrane. Therefore, through interaction with CRLF1, SorLA regulates CLCF1/CNTFRα signalling and turnover.

CRLF1, aside from the two binding sites for CLCF1 and CNTFRα, has another one for sortilin-related receptor 1 (SorLA), and it can bind all targets simultaneously. The CRLF1/CLCF1 and CRLF1/CLCF1/sCNTFRα complexes bind to SorLA via the CRLF1 subunit. Depending on the binding partner, this binding can either modulate the cellular response to CRLF1/CLCF1 by mediating CRLF1-dependent endocytosis and subsequent lysosomal degradation (membranous CNTFRα) or enhance signalling, particularly in CNTFR-deficient cells, by binding and accumulating complexes of CRLF1/CLCF1/ sCNTFRα at the cell membrane (soluble form of CNTFRα) (Figure 4). Thus, through interaction with CRLF1, SorLA regulates CLCF1/CNTFRα signalling and turnover [30].

The CRLF1/CLCF1 complex also binds Sortilin, and this facilitates GP130/LIFRβ-mediated signalling by interacting with LIFRβ and, e.g., increasing its affinity for ligands promoting the assembly of the GP130/LIFRβ heterodimer [31]. Sortilin, also known as neurotensin receptor-3, and the sorting-related receptor with type-A repeats (SorLA) are members of the Vps10p domain receptor family. SorLA has recently been implicated in the immune response by regulating IL-6-mediated signalling and driving monocyte migration. Sortilin acts as a receptor, co-receptor, and intra- and extracellular trafficking regulator [32].

### 2.5. CRLF1/p28 Complex

**Figure 5:**
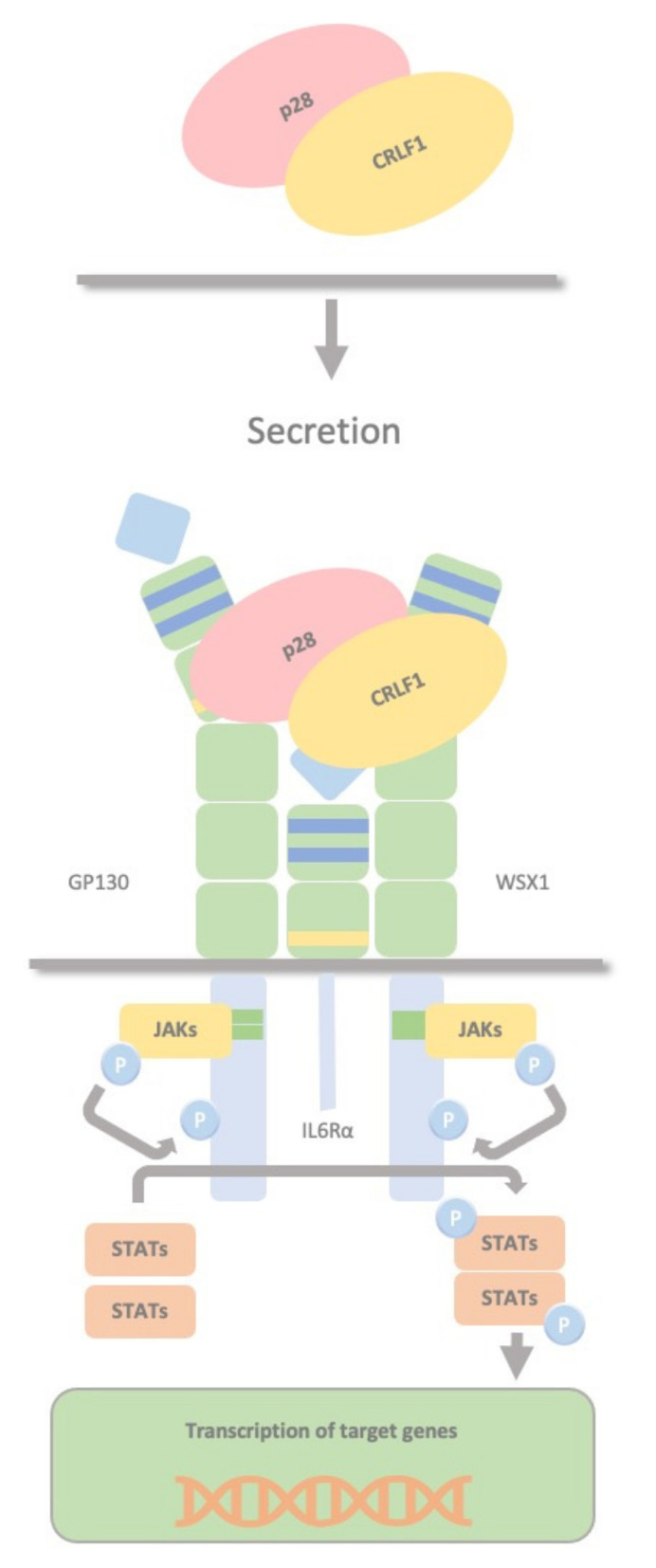
CRLF1/p28 complex. The IL-27 complex is formed by the association of the cytokine p28 with the soluble cytokine receptor EBV-induced gene 3 (EBI3). CRLF1 can chaperone the secretion of p28 to form a third composite cytokine that binds and signals through a different complex containing GP130, the WSX1 receptor and the interleukin 6 receptor (IL-6R). This also activates STAT1 and STAT3 phosphorylation and possibly SHP2/Ras/MAPK signalling within the target cell. This complex is produced by dendritic cells, stimulates NK cells, and inhibits CD4 T cell proliferation, highlighting a role in regulating NK and T cell functions by dendritic cells.

CRLF1 can also chaperone the secretion of p28 to create a composite cytokine that binds and signals through a different complex, the IL27R, formed by GP130, the WSX1 receptor and the interleukin 6 receptor (IL-6R). The IL-27 complex derives from the association of the cytokine p28 with the soluble cytokine receptor EBV-induced gene 3 (EBI3). This complex activates STAT1 and STAT3 phosphorylation and possibly SHP2/Ras/MAPK signalling within the target cell (Figure 5). Dendritic cells produce this complex, which stimulates NK cells, and inhibits CD4 T cell proliferation, highlighting the role of dendritic cells in regulating NK and T cell functions. P28 does not require CRLF1 to initiate signalling through this receptor complex; therefore, CRLF1 acts only as a chaperone in this case [33]. Furthermore, p28/CRLF1 is active on mouse B cells and induces plasma cell differentiation [34].

## 3. CRLF1 and CLCF1 in Development, Health and Disease

### 3.1. CS/CISS1 and CS/CISS2

Mutations in *CRLF1* or *CLCF1* are associated with Crisponi/cold-induced sweating syndrome (CS/CISS). More than 40 disease-causing mutations in *CRLF1* (CS/CISS1, MIM# 601378) have been identified in 96 CS/CISS1 individuals [35,36,37]. In contrast, only three cases have been described with pathogenic mutations in *CLCF1* associated with CS/CISS2 (MIM# 610313, [38,39]). CS/CISS’s main neonatal clinical features include febrile crises with temperatures up to 42 °C (hyperthermia), orofacial/laryngeal muscle constrictions, including paroxysmal contractions in the facial and oropharyngeal muscles, dysphagia, and camptodactyly. In adolescence, hyperthermia spontaneously improves; cold-induced sweating develops at ambient temperatures below 20 °C.

Studies of fibroblasts from CS/CISS1 individuals with mutations in *CRLF1* showed that STAT3 phosphorylation, following activation of this pathway, was normally triggered by LIF [36]. Moreover, the complexes of CLCF1 and wild-type or mutated forms of CRLF1 associated with CS/CISS1 could elicit STAT3 phosphorylation in an IMR32 cell line expressing the CNTFR complex while CRLF1 alone cannot [29]. Similar studies conducted in SK-N-GP cells expressing the CNTFR complex showed that wild-type CLCF1 alone could induce STAT3 phosphorylation while the mutant R197L CLCF1 associated to CS/CISS2 failed [38].

Therefore, although CRLF1 is not necessary to activate the CNTFR pathway, its secretion appears to be required for proper CLCF1 secretion and adequate activation of the CNTFR pathway since its impairment is associated with CS/CISS in humans.

### 3.2. Nervous System

CS/CISS’s most common clinical feature is orofacial/laryngeal muscular spasms, reported in 83% of CS/CISS individuals [37]. Studies using *Crlf1* and *Clcf1* knockout mice show that pups lacking CRLF1 or CLCF1 have no observable abnormalities at birth but fail to suckle and die within 24 h after birth [24,40], suggesting that both CRLF1 and CLCF1 are necessary for the recognition or processing of pheromonal signals or for the mechanics of suckling itself. Detailed studies of knockout animals showed that both CRLF1 or CLCF1 loss of function is associated with reduced numbers of motor neurons in the facial nucleus and ventral horn of the lumbar spinal cord. *Crlf1* and *Clcf1* mRNA was found in skeletal muscle fibres of embryonic mice during the motoneuron cell death period, between embryonic day (E)14.5 and E18.5 of mouse development. These findings support the view that the CRLF1/CLCF1 complex is a target-derived factor required to survive specific pools of motoneurons. CLCF1 can rescue motoneurons when provided exogenously [41]. Motoneurons are involved in the contraction and relaxation of muscles, and severe contractions of the facial muscles and orofacial weakness are characteristic features of CS/CISS. Interestingly, in this respect, mutations in CRLF1 have also been found in patients with familial achalasia, suggesting a role of CRLF1 signalling in the relaxation of the lower oesophageal sphincter [42].

Moreover, CLCF1 can sustain embryonic motor and sympathetic neurons in vitro [21,26], promote astrocyte differentiation in neural stem cells [43], and has emerged as a prominent neuropoietic cytokine. CRLF1 added to conditioned medium for the induction of neuroepithelial cells can overcome the weak activity of CLCF1 in inducing astrocytes [43]. *Clcf1* is expressed in a subpopulation of vasopressin neurons in the dorsal suprachiasmatic nucleus in the hypothalamus, with a putative role in the circadian control of mammalian locomotor activity [44].

Seventy-three percent of CS/CISS individuals showed severe thermoregulatory abnormalities, including hyperthermia without infections up to 42 °C [37]. Thermoregulatory processes are essential for maintaining body homeostasis and are controlled by several levels of the nervous system. Thermoreceptors of the skin register temperature fluctuations of the environment and transmit this temperature information via somatosensory neurons to the central nervous system [45,46]. Thermosensitive neurons (heat- and cold-sensitive neurons) of the hypothalamus regulate the body temperature via reciprocal heat-emitting or heat-generating mechanisms [46]. The cause of the severe thermoregulatory complications in the neonatal period in CS/CISS is not entirely understood.

Besides the thermoregulatory abnormalities in the neonatal period, adolescent CS/CISS individuals show cold-induced sweating at temperatures below 20 °C with age at onset > 6 years old [35]. The CRLF1/CLCF1 complex, acting through the CNTF receptor, is essential for cholinergic differentiation of sweat gland innervation [47,48]. During development, some sympathetic neurons innervating different target tissues, such as sweat glands, periosteum and vasculature, switch from noradrenergic to a cholinergic phenotype depending on the interaction of the neurons with the target tissue. Treatment of neonatal rats with the neurotoxin 6-hydroxydopamine (6-OHDA) exerts neurotoxic effects primarily on catecholaminergic neurons, where it can cause oxidative damage and subsequent selective destruction of noradrenergic terminals in the peripheral nervous system [49,50]. Such treatment provided evidence for a transition from noradrenergic to cholinergic phenotype during the development of sympathetic neurons innervating both sweat glands [51] and periosteum [52,53]. In mice, sympathetic neurons that contact the developing sweat glands or the periosteal covering of the sternum during embryonic development are noradrenergic: they express tyrosine hydroxylase (TH) and dopamine β-hydroxylase (DBH) and produce norepinephrine (NE; [54]). However, from approximately postnatal day P4 and up to P21, there is a gradual loss of these noradrenergic markers and acquisition of the cholinergic markers choline acetyltransferase and acetylcholinesterase, as well as the neuropeptides, vasoactive intestinal peptide (VIP), and calcitonin gene-related peptide [52,54,55,56,57]. At early P21, the density and distribution of sympathetic cholinergic fibres are consistent with those observed in adults. The similarities in the transmitter properties of the mature sympathetic innervation of the periosteum and sweat glands are striking [52]. The complex affecting this switching is likely related or identical. Furthermore, signalling through GP130 is essential for the postnatal induction of cholinergic properties in previously noradrenergic neurons innervating mammalian sweat glands, as demonstrated by GP130 mutational inactivation in mice [58] and by receptor blockade in cultures of rat sympathetic neurons [59]. The absence of the sympathetic switch in CS/CISS can explain the impaired thermoregulation and abnormal sweating that accompanies affected individuals throughout their lives [35].

Other observations indicate the involvement of CRLF1 and CLCF1 in neural repair mechanisms. mRNA levels of *CLCF1*, *CRLF1*, *CNTFR*, *LIF*, and *LIFR* are increased after optic nerve injury in zebrafish [60] and sciatic nerve injury in mice [61]. Silencing *clcf1* or *crlf1* genes in zebrafish retinal explants suppressed optic axon regrowth [60], indicating that CLCF1/CRLF1 may enhance optic nerve regeneration in mammals.

Neuroblastoma SH-SY5Y cells change their susceptibility to oxidative stress during differentiation [62]. A comparative analysis of gene expression between undifferentiated and differentiated neuroblastoma cells after 6-OHDA treatment identified CRLF1 as a putative mediator of oxidative stress resistance [63]. Interestingly, CRLF1 does not require CLCF1 or GP130 in this novel role, although the studies were performed only in a tumour-derived cell model system. In contrast to other cells, in neurons, exposure to agents that increase oxidative stress inhibits JAK-mediated signalling [64]. Further studies will help better understand the underlining mechanism and whether recombinant CRLF1 may be helpful in neuroprotective therapies.

### 3.3. Kidney

In one recent CS/CISS1 report, the patient presented with urinary system complications, including a small ectopic nonfunctioning right kidney and persistent mild hypernatremia, suggesting a possible role of CRLF1 in renal development [65]. Knowledge about the expression of *CRLF1* and *CLCF1* in developing and adult kidneys is sparse. The first expression data from Northern blotting assays go back to the first descriptions of CRLF1 and CLCF1. *Crlf1* is primarily expressed in developing kidneys and less in adults [24], while *Clcf1* is mainly expressed in adult kidneys and barely detectable in developing kidneys [21,22]. *Crlf1* expression levels are elevated in the E12.5 embryonic kidney compared to adults [66]. The mesonephric (Wolffian) duct at E12.5 and the growing tips of the collecting ducts of the kidney express *Crlf1* throughout embryogenesis [24].

The Genotype-Tissue Expression (GTEx) project [67] reports lower *CRLF1* expression levels in adult human kidneys than *CLCF1* by RNA seq. The Human Protein Atlas project [68], in particular, the single-cell type transcriptomics map of human tissues, also reports a higher *CLCF1* expression compared to *CRLF1* in adult kidneys; *CLCF1* is mainly expressed in proximal and collecting duct cells (https://www.proteinatlas.org/ENSG00000175505-CLCF1/single+cell+type/kidney, accessed on 22 December 2021), while *CRLF1* (https://www.proteinatlas.org/ENSG00000006016-CRLF1/single+cell+type/kidney, accessed on 22 December 2021) in distal tubular cells [69]. Data are available from version 21.0 proteinatlas.org (accessed on 22 December 2021).

Kidney development in mammals is a highly orchestrated multistage process depending on precise and complex interactions among different compartments. Disturbance of this process results in renal anomalies. The main steps foresee three spatially and temporally distinct kidneys, the pronephros, mesonephros, and metanephros. The definitive kidney, the metanephros, begins forming when metanephric mesenchyme (MM) cells induce outgrowth of the ureteric bud (UB) from the nephric duct at E10.5 in mice (weeks 4–5 of gestation in humans). Afterwards, the UB secretes signals that induce the MM to condense around the growing UB tip, forming the cap mesenchyme (CM). At this stage begins CM-guided UB branching morphogenesis that occurs rapidly until mid-gestation and slows down after E15.5 [70,71]. Gene expression studies conducted in mice for the identification of such signals found that one of the genes that were highly specific to the UB tip was *Crlf1* [72,73,74] with strong expression in mouse (E15.5) and humans (week 15–16; [75]). The expected growth and morphogenesis of the UB during kidney development requires several intracellular signalling pathways activated by the glial cell-derived neurotrophic factor (GDNF) through the Ret receptor tyrosine kinase, including Ras-ERK mitogen-activated protein (MAP) kinase, Phosphatidylinositol-3 kinases (PI3K)-Akt pathways, and others. These signals lead to changes in gene expression in UB tip cells. Among the GDNF-induced genes, *Crlf1* was found as part of a regulatory network that promotes UB branching morphogenesis and other aspects of kidney development [76].

Furthermore, *Crlf1*, along with other genes promoting epithelial-to-mesenchymal transition and fibrosis, has been found up-regulated in the kidney of krüppel-like zinc finger protein Gli-similar 2 (*Glis2*) mutant mice, showing severe renal atrophy and fibrosis starting at 8 weeks of age [77], with a nephronophthisis (NPHP)-like phenotype in humans and mice. NPHP is an autosomal recessive cystic kidney disease, which represents the most frequent genetic cause for end-stage renal disease in the first three decades of life. Furthermore, recombinant CRLF1 in complex with CLCF1 triggered phosphorylation of STAT3 in rat-isolated metanephric mesenchyme, a pathway characteristic of mesenchymal-to-epithelial conversion. Again, when applied to isolated rat metanephric mesenchyme, CRLF1/CLCF1 induced mature nephron structures expressing glomerular and tubular markers [72]. However, considering that developing kidneys barely express CLCF1, it could be that an alternative ligand mediates this developmental action of CRLF1. CLCF1 and others have been identified as potential circulating permeability factors in the plasma of patients with focal segmental glomerulosclerosis (FSGS), a clinicopathological syndrome characterized by nephrotic-range proteinuria with a high incidence of progression to end-stage renal disease [78,79,80,81]. It is still unclear whether these factors are truly pathogenic, specific and how many combinations of circulatory factors exist [82].

Further studies in mice showed that FSGS serum and CLCF1 increased albumin permeability (Palb) and altered the actin cytoskeleton of podocytes through the JAK2/STAT3 pathway [78]. These alterations can be blocked/decreased by an anti-CLCF1 monoclonal antibody, heterodimer CRLF1/CLCF1, JAK2 inhibitor and STAT3 inhibitor [79,81]. Opposite effects of heterodimer CRLF1/CLCF1 and CLCF1 contrast with the reported similarities in their effects on neuronal cells and advise cell-type specificity [81]. Increasing evidence highlights the role of the JAK/STAT signalling pathway in glomerular disease, which makes JAK and/or STAT potential targets for treating glomerular disease [83]. Regarding the JAK/STAT pathway, JAK2 and STAT3 are the predominant isoforms in glomeruli and podocytes of mice and rats [78]. This evidence suggests that antibodies targeting CLCF1 or its receptors and the JAK/STAT pathway inhibitor may be novel therapeutic targets for primary FSGS and recurrent FSGS.

### 3.4. Bone and Cartilage

CS/CISS individuals present with skeletal abnormalities, including spinal kyphoscoliosis, palatal and frontonasal malformations, jaw malformations, elbow contracture and camptodactyly. *Crlf1* and *Clcf1* are expressed in the developing murine skeleton, both in limb buds [23,24] and embryonic muscle and cartilage [41]. In situ hybridization in mouse embryos at E12.5 showed *Crlf1* expression in all pre-cartilaginous and membranous blastema. Moreover, at E14.5, *Crlf1* expression was present in the pre-cartilaginous condensations of the digital metacarpals, in the intervertebral discs and facial mesenchyme [24]. *Crlf1* was also detected by in situ hybridization in hypertrophic chondrocytes, osteoblasts, and osteocytes in a mouse model of ectopic bone formation [84]. CLCF1 and CRLF1 were detected in cultured murine primary osteoblasts. PTH parathyroid hormone [85,86], a hormone that stimulates bone formation depending on GP130 signalling within the osteoblast lineage, stimulates their transcription [87]. Recently, CRLF1 was downregulated in bone subjected to mechanical loading [88]. These data suggest a role for CRLF1 and CLCF1 in bone formation.

Bone is a dynamic tissue that constantly remodels throughout life, providing mechanical support for stature and locomotion and protecting vital organs such as bone marrow and the brain [89]. Bone formation relies on different cell types, osteoblasts, osteoclasts and osteocytes in bone and chondrocytes in cartilage. Osteoblasts, osteocytes, and chondrocytes arise from multipotent mesenchymal stem cells (MSCs) [90,91]. Osteoclasts derive from hematopoietic stem cells (HSCs) in the monocyte/macrophage lineage [92]. There are two significant modes of bone formation or osteogenesis, and both involve transforming a pre-existing mesenchymal tissue into bone tissue. The conversion of mesenchymal tissue into bone is called intramembranous ossification and occurs primarily in the bones of the skull. In other cases, the mesenchymal cells differentiate first into chondrocytes (chondrogenesis) and proliferate, forming the cartilage. After the hypertrophic phase, chondrocytes die by apoptosis and are lately replaced by osteoblasts forming the bone in a process called endochondral ossification [93].

Chondrocytes, osteoblasts, osteoclasts, and osteocytes rely on JAK1/STAT3/SOCS3 signalling during normal bone development. JAK1 and STAT3 transduce signals initiated by the IL-6 family cytokines that stimulate chondrocytes, osteoblasts, and osteoclasts by binding to the GP130 receptor and are essential for the normal skeletal development of mice and humans [94]. Deleting either *Gp130* or *Lifr* results in a neonatal lethal skeletal phenotype of low bone mass, high osteoclast number, and low osteoblast number [95,96]. Mice with osteoblast-specific disruption of the *Stat3* gene showed an osteoporotic phenotype because of a reduced bone formation rate [97]. No bone defect has been reported in mice lacking *Crlf1, Clcf1* or *Cntfrα* [24,40,98].

Bone remodelling is an active and dynamic process that occurs throughout life, and that relies on the correct balance between bone resorption by osteoclasts and bone deposition by osteoblasts. If there are too many active osteoclasts, too much bone will be dissolved, and osteoporosis will result. Conversely, if not enough osteoblasts are produced, the bones are not hollowed out for the marrow, and osteopetrosis results [99]. CLCF1 is reduced in women with postmenopausal osteoporosis [100,101]. In one study, CLCF1 alleviated bone loss in osteoporosis mouse models by suppressing osteoclast differentiation by activating interferon signalling and suppressing the NF-κB signalling pathway [102]. The receptor activator of the NF-κB ligand (RANKL) binds to RANK, the NF-κB receptor activator on myeloid lineage cells and functions as a critical factor for osteoclast differentiation and activation. Osteoprotegerin (OPG), a decoy receptor for RANKL, protects the skeleton from excessive bone resorption by binding to RANKL and preventing it from binding to its receptor, RANK. Thus, the RANKL/OPG ratio is essential for bone mass and skeletal integrity [103].

Furthermore, CLCF1 showed no adverse effects on osteoblast differentiation and function in vitro and vivo. These results appear different from other reported findings suggesting CLCF1 could suppress bone formation directly on mature osteoblasts [86,104]. The discrepancy could be due to differences in protein doses, stimulation, exposure time, and the cell types used in the experiments. Therefore, further studies are needed to precisely understand the role of CLCF1 in the bone remodelling process and osteoporosis. Nevertheless, this function of CLCF1 in bone remodelling suggests a potentially useful therapeutic role for the treatment of osteoporosis [102]. CRLF1 is expressed in osteoblasts and chondrocytes and up-regulated in SW1353 chondrosarcoma cells transduced by the key chondrogenic transcription factor SOX9 [105].

Both micrognathia and retrognathia have been reported in CS/CISS1 patients, and precise control of jaw length during development is crucial for proper form and function. *Crlf1* expression was detected in the first branchial arch at E9.5, E11.5 and E14.5 in the facial mesenchyme [24]. Mandibles develop from the first branchial arch between E10.5 and E14.5. Levels of bone resorption are inversely proportional to jaw size and essential in determining overall mandibular size between and among species [106]. The sympathetic nervous system controls bone remodelling by regulating bone formation, and resorption and different sympathetic pathways may control distinct bone envelopes. During development, the sympathetic innervation of the periosteum switches from a catecholaminergic phenotype to a cholinergic phenotype [52]. A sympathetic cholinergic system innervates the mandible periosteum, and sympathectomy decreases the number of preosteoclasts and RANKL-expressing osteoblasts [107]. In particular, the number of osteogenic cells expressing RANKL was reduced in the periosteum of sympathectomized rats, along with an increase in OPG expression. Sympathectomy impairs osteoclastic resorption along the mandible periosteum. The rise in OPG expression resulted in an imbalance in the OPG/RANKL mRNA ratio oriented to inhibition of osteoclast differentiation. This data confirms the critical role of the cholinergic sympathetic innervation and eventually of CRLF1 in regulating mandible bone development and metabolism.

Cartilage diseases such as osteoarthritis (OA), characterized primarily by progressive degradation and calcification of articular cartilage [108], represent one of the most common clinical conditions affecting the quality of life in the developed world. Cartilage is predominantly made of an extracellular matrix with a low density of cells, and matrix changes play an essential role in the progression of diseases affecting the tissue. The etiopathogenesis of OA is not entirely understood, and various factors are implicated in disease initiation and progression. In addition to mechanical damage, an altered cytokine balance favouring pro-inflammatory cytokines leads to low-grade inflammation, which is responsible for cartilage degradation, bone remodelling and synovial proliferation [108,109]. CRLF1 has been reported to promote the proliferation of chondrocytes [110].

Further studies showed that overexpression of CRLF1 inhibited bone marrow mesenchymal stem cells (BMSCs) viability, induced apoptosis, and suppressed chondrogenic differentiation of BMSCs. In addition, a substantial body of evidence exists suggesting OA significantly correlates with chondrocyte apoptosis. However, direct evidence demonstrating a causal link between chondrocyte apoptosis and OA remains to be established [111,112].

CLRF1 is expressed at high levels in osteoarthritic human knee cartilage and was up-regulated after stimulation of mouse chondrocytes by transforming growth factor-β (TGF-β; [110]. CRLF1/CLCF1 promoted the proliferation of chondrocyte precursors. It suppressed the expression of aggrecan and type II collagen, an abundant component in the cartilage ECM, in in vitro experiments using the mouse chondrogenic cell line ATDC5 [110]. *Crlf1* expression was up-regulated when ATDC5 cells were exposed to TGF-β and downregulated when exposed to bone morphogenetic protein-2 (BMP-2), tumour necrosis factor α (TNF-α), IL-6, and IL-1β. However, *Clcf1* did not show any change in expression between intact and damaged cartilage; therefore, the most influential molecule in the signalling of the CRLF1/CLCF1 complex in damaged cartilage might be CRLF1. This evidence suggests that the CRLF1/CLCF1 complex may disrupt cartilage homeostasis and promote the progress of osteoarthritis. However, its function in chondrogenic differentiation remains unclear [113]. In ectopic bone formation induced by injection of BMP-2 into the muscle, expression of *Crlf1* was induced 5-fold at day 5 after BMP-2 injection. Temporally, CRLF1 induction preceded the stage of chondrogenesis in this model of endochondral osteogenesis [84].

Furthermore, rs7256319 C > T SNP in *CRLF1* was found to have a markedly imbalanced expression of its respective alleles in articular cartilage, as reflected by consistently lower expression of the alternative allele T compared with the reference allele C in heterozygotes [114]. As previously reported [110,115], *CRLF1* appeared to be significantly up-regulated in lesioned compared to preserved OA articular cartilage (fold change 4.6, FDR = 3.1 × 10^−10^), as was its signalling partner *CLCF1* (fold change 2.1, FDR = 1.0 × 10^−6^). *CNTFR* instead was significantly downregulated (fold change 0.3, FDR = 1.9 × 10^−8^). Additionally, it was shown by Tsuritani et al. [110] that up-regulation of the CRLF1/CLCF1 complex in ATDC5 cells disrupts cartilage homeostasis and promotes progression of OA by enhancing the proliferation of chondrocytes and suppressing the production of cartilage matrix. Therefore, it was hypothesized that the alternative allele T of rs7256319 may mitigate CRLF1/CLCF1 signalling in heterozygous carriers toward ongoing cartilage degradation due to primary OA.

microRNAs (miRs) play a critical role in regulating cell proliferation and differentiation, acting as post-transcriptional regulatory factors. Some miRNAs can shape metabolism and bone formation, controlling the proliferation and differentiation of osteoblasts, osteoclasts, and chondrocytes [116]. miR-320 was recently discovered as a negative regulator of osteoblast differentiation of hMSCs [117]. Xu et al. [113] found that miR-320 is directly bound to the 3’UTR of *CRLF1*. Overexpression of CRLF1 inhibited BMSCs viability, induced apoptosis, and suppressed chondrogenic differentiation of BMSCs. Suppression of CRLF1 via activation of miR-320 promotes the chondrogenic differentiation of BMSCs and protects cartilage tissue from damage in osteoarthritis [113].

Hypertrophy of the ligamentum flavum (HLF) is one of the common causes of lumbar spinal stenosis (LSS). Many profibrotic factors can promote the secretion of CRLF1. Elson et al. [23] found that CRLF1 can be up-regulated in some fibroblasts by TNF-α, IL-6, and interferon-γ. In chondrocytes, secretion of CRLF1 was regulated by TGF-β1 [110]. In HLF, CRLF1 was abundantly expressed, whereas CLCF1 was not elevated [118]. Thus, CRLF1 might be a novel independent indicator for HLF. Profibrotic actions of both TGF-β1 and CRLF1 are mediated via the ERK signalling pathway. This investigation identified for the first time that the CRLF1-ERK pathway is involved in HLF downstream of TGF-β1, suggesting that CRLF1 may act as an independent regulator to promote the pathogenesis of HLF formation via the ERK signalling pathway, and suppression of CRLF1 expression can decrease HLF formation. Therefore, CRLF1 is a crucial regulator in the pathogenesis of HLF, which offers potential strategies for the prevention and treatment of LSS [118].

### 3.5. Lung

*Crlf1* was highly expressed in the foetal lung, in lung buds and bronchi in the period of branching morphogenesis (E12.5–E18.5) [23,24] but not in the adult lung. Many CS/CISS1 individuals die in infancy with respiratory abnormalities, but lung disease has not been clearly defined [35]. *Crlf1* or *Clcf1* knockout mice die on postnatal day 1 because of a suckling defect, but no pulmonary phenotype has been described.

*CRLF1* was among the most highly up-regulated gene in idiopathic pulmonary fibrosis (IPF), a progressive and typically fatal lung disease, compared with normal controls [119]. *CRLF1* expression in the lung was restricted to type II alveolar epithelial cells and macrophages. *CNTFR* was localized mainly to type II alveolar epithelial cells and somewhat in airway epithelial cells. Type II alveolar epithelial cells are preferentially stimulated by exposure to CRLF1/CLCF1 as assessed by STAT3 phosphorylation. Furthermore, increased expression of inflammatory genes and decreased expression of several extracellular matrix genes occurred after CRLF1 administration in murine lungs. The localization of *CRLF1* expression in hyperplastic type II alveolar epithelial cells and the ability of CRLF1/CLCF1 to stimulate inflammation in the lung suggest that CRLF1 secretion may represent a generalized response to any lung injury. In addition, CRLF1/CLCF1 reduced pulmonary fibrosis, and further investigation is needed to determine whether CRLF1 has therapeutic potential as an antifibrotic agent [119]. The effect of CRLF1 in the lung is likely complex and involves Th1 and Treg lymphocytes and the production of cytokines (e.g., IFN-) and multiple chemokines [119]. IPF and lung cancer share common risk factors, epigenetic and genetic alterations, activation of similar signalling pathways, and poor survival. Studies of gene expression profiles of stromal cells from patients with IPF and lung adenocarcinoma (LUAD) and normal lung found *CRLF1* among the fourteen genes up-regulated in both IPF and LUAD to controls [120].

### 3.6. Haematopoiesis and Immune cell Function

Studies in *Crlf1* knockout mice showed that haematopoiesis was perturbed. Although the haematocrit, the number of circulating platelets, and the number and morphological distribution of white blood cells were expected, the number and lineage of haemopoietic progenitor cells in neonatal mice were generally reduced in the bone marrow and spleen of neonatal mice [24]. In support of a potential role as a haemopoietic regulator, *Crlf1* is expressed by many stromal cell lines, which are known to support hemopoiesis [24].

Haemopedia [121] is a database of gene expression profiles from a broad spectrum of haematopoietic cells to include RNA-seq gene expression data from mice and humans [122]. RNA-seq data set covers a wide range of lineages and progenitors, with 57 mouse blood cell types (flow-sorted populations from healthy mice) and 12 human blood cells. According to Haemopedia, there is a −0,37 overall Pearson correlation between *Crlf1* and *Clcf1* mouse expression (https://www.haemosphere.org/expression/genevsgene?gene1=ENSMUSG00000007888&gene2=ENSMUSG00000040663&datasetName=Haemopedia-Mouse-RNASeq (accessed on 22 December 2021)), and their expression is different between hematopoietic cell types or lineage. *Crlf1* is less expressed than *Clcf1. Crlf1* is mainly expressed in erythrocyte and B cell lineages (https://www.haemosphere.org/expression/show?geneId=ENSMUSG00000007888 (accessed on 22 December 2021)). In contrast, *Clcf1* is primarily expressed in NK, T, and dendritic cell lineages, with the lowest expression in erythrocytes and megakaryocyte lineages (https://www.haemosphere.org/expression/show?geneId=ENSMUSG00000040663 (accessed on 22 December 2021)).

Haematopoiesis entails the generation of hematopoietic stem cells (HSCs), the proliferation and maintenance of multipotential progenitors, and lineage commitment and maturation. Embryonic haematopoiesis in vertebrates occurs at various locations and appears in three successive stages (“waves”). The first hematopoietic cells of embryonic origin are present in the yolk sac (YS), starting at E7.5 of mouse embryogenesis. The spleen is a hematopoietic organ in mice. Hematopoietic stem cells (HSCs) migrate into the spleen/foetal liver around E14 and then migrate into the bone marrow (BM) around E17. Around the time of birth, the site of erythropoiesis switches to the bone marrow and spleen. In humans, erythropoiesis in adulthood is mainly dependent on the bone marrow, but in mice, the spleen remains a vital erythropoietic organ during adult life. In the vertebrate bone marrow, two types of stem cells coexist—HSCs and MSCs. Haematopoiesis occurs when these two types of stem cells and their descendants interact. The descendants of HSCs supply the body with all the mature blood cells, while MSCs give rise to stromal cells that form a niche for HSCs and regulate the process of haematopoiesis [123].

While inactivating CRLF1, which alters CLCF1 secretion, leads to a marked reduction in bone marrow (BM) progenitor cells, CLCF1 exerts immunomodulatory functions such as supporting B-cell expansion and humoral responses producing Ab with a preference of Th2 over Th1 Ig types [124]. CLCF1 overexpression in mice leads to splenomegaly, whereas mice heterozygous for CLCF1 display lower circulating leukocyte counts [124]. CLCF1 also has an influential role on hematopoietic multipotent progenitor cell proliferation in vitro with a bias towards myeloid cell differentiation [125]. In mice, CLCF1 induces B-cell expansion, enhances humoral responses and triggers autoimmunity. Immunoregulatory functions for CLCF1 are also revealed by CLCF1 injections in mice showing a significant increase in circulating pro-inflammatory monocytes [126].

Cytokine p28 and the soluble cytokine receptor EBI3 compose the Interleukin-27 [124,127]. Cytokine p28 can form an alternative composite cytokine with the EBI3 homologue cytokine-like factor 1 (CRLF1, [34]). The p28/CRLF1 complex modulates NK cell activity, and CD4 T cell cytokine production in vitro induces STAT3 phosphorylation and supports B9 plasmacytoma cell proliferation, suggesting it might be a plasma cell trophic factor. p28/CRLF1 induces IgM, IgG2c, and IgG1 production and plasma cell differentiation. It can contribute to B and plasma cell function, differentiation, and proliferation in normal and pathological conditions such as Castleman’s disease and multiple myeloma [34]. Paediatric myelodysplastic syndrome (MDS) represents a spectrum of disorders ranging from aplasia (RCC) to myeloproliferation with an increased risk of leukemic transformation (RAEB(t); [128]). CRLF1 is significantly increased in MSCs derived from children with RAEB. It remains to be elucidated whether this finding is a cause or a consequence of the disease [129].

These observations strongly suggest underexplored immunoregulatory roles for both CLCF1 and CRFL1.

### 3.7. Cancer

Lung cancer is the leading cause of cancer-related death worldwide. Non-small cell lung cancer (NSCLC) accounts for 85–90% of cases, and lung adenocarcinoma (LUAD) is the most common subtype. Approximately 30% of patients with LUAD harbour a mutation in KRAS. A large body of literature supports the hypothesis that the tumour microenvironment (TME), the so-called cancer-associated fibroblasts (CAFs), supports tumour progression by a variety of mechanisms [130]. CLCF1 is expressed by mouse CAFs, promoting tumour growth [131]. Some authors have studied the functional significance of the CLCF1/CNTFR signalling axis in LUAD. They found that the knockdown of CNTFR in mouse lung tumour cells decreases tumour growth. Subsequently, they generated a high-affinity soluble receptor (eCNTFR–Fc) that can bind CLCF1 with high affinity while simultaneously reducing its affinity for LIFR and GP130. This new molecule sequesters CLCF1, thereby inhibiting its oncogenic effects. Abrogation of CLCF1 through eCNTFR–Fc appears most effective in tumours driven by oncogenic KRAS. Overall, blockade of CLCF1/CNTFR signalling could be a novel therapeutic opportunity for LUAD and potentially for other tumour types in which CLCF1 is present in TME [132].

CAFs are critical players in multicellular, stromal-dependent alterations leading to hepatocellular carcinoma (HCC) pathogenesis. More than 80% of HCCs are characterized by extensive liver fibrosis caused by the activation, proliferation, and accumulation of fibroblasts. A hallmark feature of the tumour microenvironment (TME) of HCC is the mass of CAFs, which has been extensively reported to influence HCC progression. CAFs strongly express CLCF1. CLCF1 increased chemokine CXCL6 and TGF-β secretion in tumour cells, activated the ERK1/2 signalling of CAFs to produce more CLCF1, thus forming a positive feedback loop to accelerate HCC progression [133].

Papillary thyroid carcinoma (PTC) is one of the most common types of endocrine cancer and has a heterogeneous prognosis. PTC tissues exhibited higher *CRLF1* expression at mRNA and protein levels than the normal thyroid tissues. High *CRLF1* levels were associated with aggressive clinicopathological features and poor disease-free survival rates. CRLF1 increased cell migration and invasion in vitro and promoted growth tumours in vitro and in vivo. In addition, CRLF1 induced epithelial–mesenchymal transitions. Overexpression of CRLF1 activated the ERK1/2 and AKT pathways. These results indicate that CRLF1 enhances cell proliferation and metastasis in PTC and thus may therefore be a potential therapeutic target for PTC [134].

Colorectal cancer (CRC) is one of the most common malignancies globally. CRLF1 expression was decreased in CRC, and overexpression of CRLF1 inhibited the stemness and metastasis of CRC cells in vitro and in vivo [135]. These studies suggest that CRLF1 plays a tumour suppressor role in colorectal cancer progression, inhibiting tumorigenesis and metastasis. However, the regulatory mechanism of CRLF1 in stemness inhibition needs to document for further details on references.

**Figure 6:**
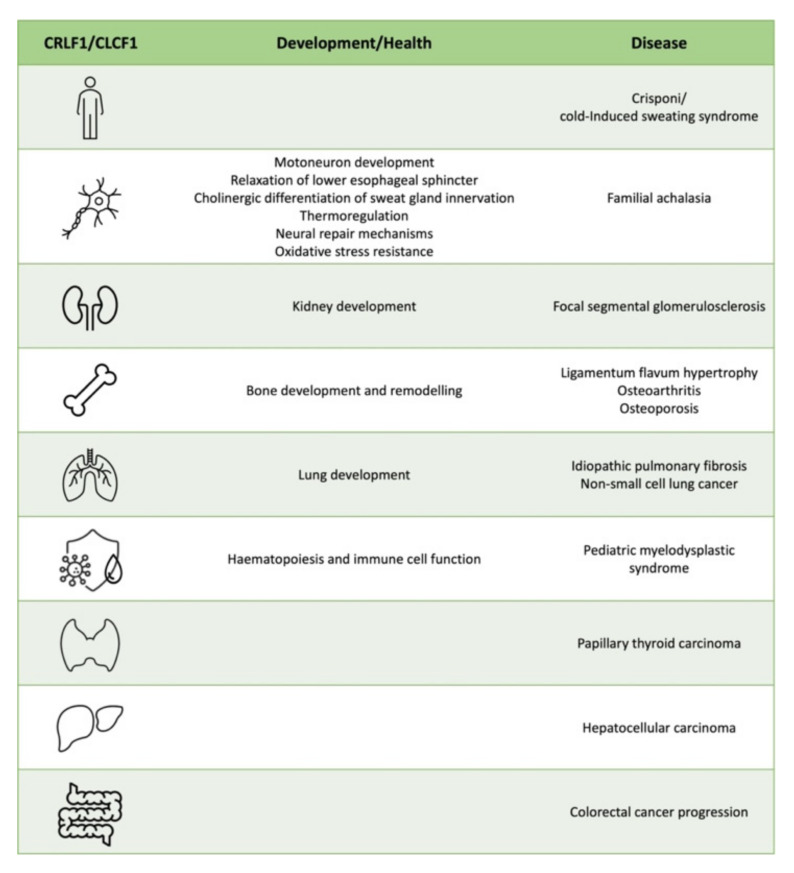
Schematic representation of CRLF1/CLCF1 function in different organs in development, health and disease.

Figure 6 shows a schematic representation of CRLF1/CLCF1 function in different organs in development, health and disease.

## 4. Therapeutic Opportunities

As summarized above, soluble cytokine receptors influence the biological functions of their ligands in different ways. The majority of the known soluble cytokine receptors have agonistic functions. They compete with their membrane-bound counterparts for the binding of their ligand. Once the cytokine is bound to the soluble receptor, it is biologically inactivated and can no longer activate its target cells. However, there are also soluble receptors that act antagonistically, thereby enhancing the effectiveness of their ligand. This interplay between soluble and cellular variants of the same cytokine receptors is possibly an endogenous mechanism to balance and control cytokine activity. This mechanism can be exploited for therapeutic approaches to modulate cytokine activity. As described in several examples above, various soluble cytokine receptors have been recombinantly produced. They are currently used in different stages of (pre) clinical studies to modulate cytokine activity to alleviate diseases [9].

Other therapeutic options include small molecule therapies that affect cytokine receptor signalling through the receptor itself or the JAK/STAT pathway [136]. Although the JAK/STAT signalling mechanism is quite simple, the biological effects of this pathway are complex because of its interaction with other signalling pathways. Detailed elucidation of JAK/STAT signalling mechanisms in disease pathogenesis is crucial to provide essential clues for the clinical treatment of diseases [137]. Since the underlying disease-causing mechanisms are often not sufficiently understood, treatment of affected patients is usually limited to symptomatic therapy. In this respect, CS/CISS patients are usually treated with agonists of imidazoline type 1 receptors, moxonidine or clonidine, to reduce paradoxical sweating [27].

## 5. Conclusions and Outlook

Considering the pivotal role that cytokines and their receptors, either transmembrane or soluble, play in health and disease, they are all promising candidates for therapeutic interference [138].

Recombinant cytokines have generally not been widely used therapeutically, with some exceptions. Although rare, long-term treatment with recombinant cytokines can result in endogenous antibodies against the cytokine [6]. In general, the pleiotropic nature of many cytokines may result in unpredictable and intolerable inflammation-associated side effects, limiting the use of recombinant cytokines in the clinic [139].

Since the identification of CRLF1 and CLCF1 and the involvement of CS/CISS, there has been a growing understanding of their pleiotropic roles in development, health and other diseases. There is still much to learn about their role in biological function and the potential therapeutic applications of these cytokines. However, most of this knowledge comes from sparse studies, mostly microarray and transcriptional studies, in various pathological conditions. Further studies are needed to unravel their specific functions and underlying physiopathological mechanisms and signalling pathways to develop knowledge-based therapies that could help treat the rare and more common diseases they are involved in.

## Data Availability

The data presented in this study were obtained from the: -GTEx Portal (https://www.gtexportal.org/home/) GTEx Analysis Release V8 (dbGaP Accession phs000424.v8.p2) accessed on 22 December 2021; -Haemopedia (https://www.haemosphere.org), Version 4.9.5 released (February 2019) accessed on 22 December 2021; -The Human Protein Atlas (https://www.proteinatlas.org/), Version 21.0 proteinatlas.org, accessed on 22 December 2021.
